# GC-MS Analysis and Microbiological Evaluation of Caraway Essential Oil as a Virulence Attenuating Agent against *Pseudomonas aeruginosa*

**DOI:** 10.3390/molecules27238532

**Published:** 2022-12-03

**Authors:** Mona Fekry, Galal Yahya, Ali Osman, Mohammed W. Al-Rabia, Islam Mostafa, Hisham A. Abbas

**Affiliations:** 1 Department of Pharmacognosy, Faculty of Pharmacy, Zagazig University, Zagazig 44519, Egypt; 2 Department of Microbiology and Immunology, Faculty of Pharmacy, Zagazig University, Zagazig 44519, Egypt; 3 Department of Biochemistry, Faculty of Agriculture, Zagazig University, Zagazig 44511, Egypt; 4 Department of Microbiology and Medical Parasitology, Faculty of Medicine, King Abdulaziz University, Jeddah 21589, Saudi Arabia

**Keywords:** caraway, essential oil, GC-MS, *Pseudomonas aeruginosa*, quorum sensing, bacterial biofilm

## Abstract

The emergence of resistant microbes threatens public health on our planet, and the emergence of resistant bacteria against the most commonly used antibiotics necessitates urgent alternative therapeutic options. One way to fight resistant microbes is to design new antimicrobial agents, however, this approach takes decades of research. An alternative or parallel approach is to target the virulence of bacteria with natural or synthetic agents. Active constituents from medicinal plants represent a wide library to screen for natural anti-virulence agents. Caraway is used as a traditional spice and in some medicinal applications such as carminative, antispasmodic, appetizer, and expectorant. Caraway essential oil is rich in terpenes that were previously reported to have antimicrobial activities. In our study, we tested the caraway essential oil in sub-inhibitory concentration as a virulence agent against the Gram-negative bacteria *Pseudomonas aeruginosa*. Caraway essential oil in sub-inhibitory concentration dramatically blocked protease activity, pyocyanin production, biofilm formation, and quorum sensing activity of *P. aeruginosa*. The gas chromatography–mass spectroscopy (GC-MS) profile of caraway fruit oil identified 13 compounds representing 85.4% of the total oil components with carvone and sylvestrene as the main constituents. In conclusion, caraway essential oil is a promising virulence-attenuating agent that can be used against topical infections caused by *P. aeruginosa*.

## 1. Introduction

Bacterial resistance has been developed against available antimicrobial agents, and multi-drug and pan-antibiotic resistance are frequently detected for several microbes. e.g., *P. aeruginosa*, *K. pneumoniae*, *E. coli*, *S. flexneri*, and *A. baumannii* [1,2,3,4,5,6]. Major causes of antibiotic resistance are an improper use of antibiotics on one hand, and resistance emergence developed by the microbes on the other hand [7,8,9]. One strategy utilized by bacteria to limit the uptake and impair the penetration of antibiotics is the formation of biofilm [10,11,12].

Biofilm is a slimy polymeric matrix formed by clusters of microorganisms attached to non-biological surfaces in response to environmental stresses [13,14,15,16,17,18]; biofilms represent protective shields for microbes against antibiotics [19,20]. Inside these matrices and inside microenvironments of high cell density in general, bacterial cells communicate through a quorum sensing system that regulates gene expression regulating biofilm formation [21].

A novel approach to combat antimicrobial resistance in general and bacterial biofilms, in particular, is the application of essential oils extracted from medicinal plants. Medicinal plants containing essential oils usually have antimicrobial and anti-fungal activity because of their active constituents [22,23,24]. The essential oils exhibit their antimicrobial activity mostly through penetration of the bacterial cell membrane leading to cell lysis, and they can prevent or disrupt bacterial biofilm through compromising cell contact or surface attachment [25,26]. In addition, they exert synergistic effects with antibiotics that are useful to overcome multi-drug-resistant pathogens [3]. 

Caraway (*Carum carvi*, family *Apiaceae*) is a biennial herb with a pleasant flavor. It is used as a spice, carminative, antispasmodic, appetizer, expectorant, and antidote for venomous beats. It is used for the treatment of digestive disorders, anemia, weight loss, eczema, and animal scabies [27,28,29]. Carvacrol, limonene, carvone, carvenone, α-pinene, linalool, γ-terpinene, and p-cymene are considered major constituents of its oil. Moreover, caraway contains flavonoids such as quercetin-3-glucuronides, quercetin 3-O-caffeyl glucoside, isoquercitrin, and kaempferol 3-glucoside [28]. 

Caraway essential oil has antioxidant, anticancer, hepatoprotective, nephroprotective, antiulcerogenic, spasmolytic, insecticidal, anticonvulsant effects, and antimicrobial activities. The antimicrobial activity of caraway essential oil has been reported against *A. niger*, *S. aureus*, *C. albicans*, *S. enterica*, *E. coli,* and *Penicillium* spp. [30,31]. Additionally, the caraway essential oil has antimicrobial activity against *P. aeruginosa*, *B. mallei*, *B. abortus,* and *B. bronchiseptica*. *P. aeruginosa* (a Gram-negative bacteria) is the causative agent for several eye, wound, and respiratory tract infections due to its various virulence factors [32,33,34,35]. *P. aeruginosa* strains constitute about 13% of drug-resistant strains. Formation of biofilms and low membrane permeability are major mechanisms of resistance to antibiotics in *P. aeruginosa* [36,37,38,39]. 

In this study, we defined the chemical composition of the investigated oil using GC-MS analysis. Moreover, we evaluated the antioxidant activity and the antimicrobial activity of freshly extracted caraway essential oil against *P. aeruginosa*, we tested the virulence mitigation activity of caraway oil regarding biofilm inhibition, pyocyanin production, protease activity, and quorum sensing. 

## 2. Results

### 2.1. Caraway Essential Oil Composition and Antioxidant Activity

**Figure 1:**
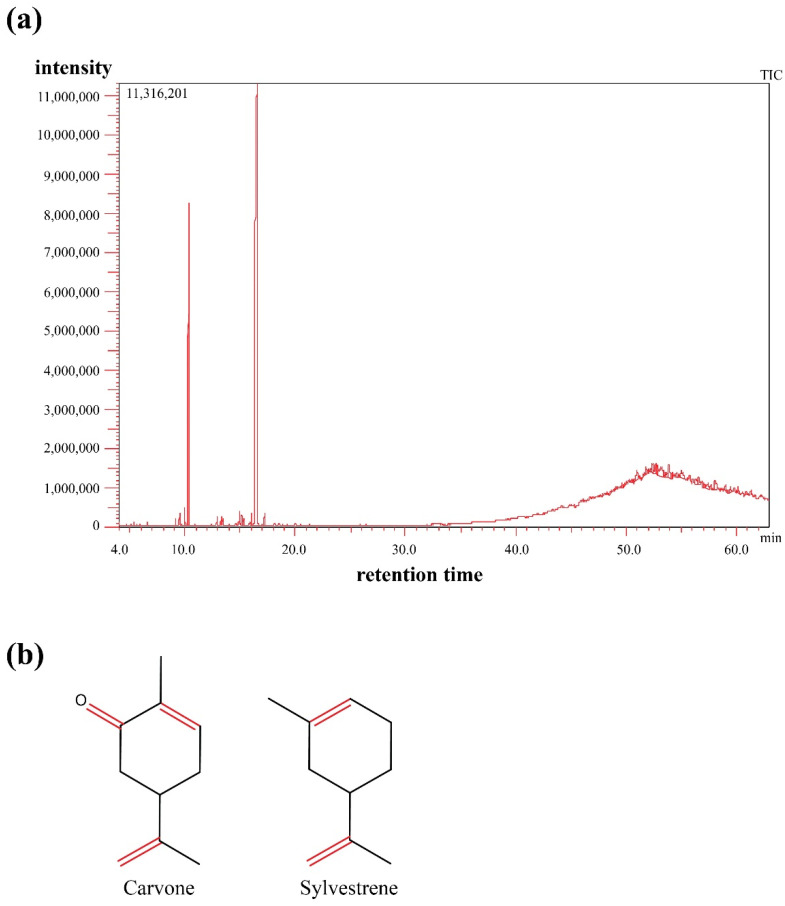
GC-MS profile of caraway fruits essential oil in (**a**) and major components (carvone and sylvestrene) in (**b**).

Thirteen compounds covering 85.4% of the total essential oil contents were identified using GC-MS (Figure 1a). Carvone and sylvestrene represented the major oil components with 63.7% and 14.8%, respectively (Figure 1b and Table 1). 

**Figure 2:**
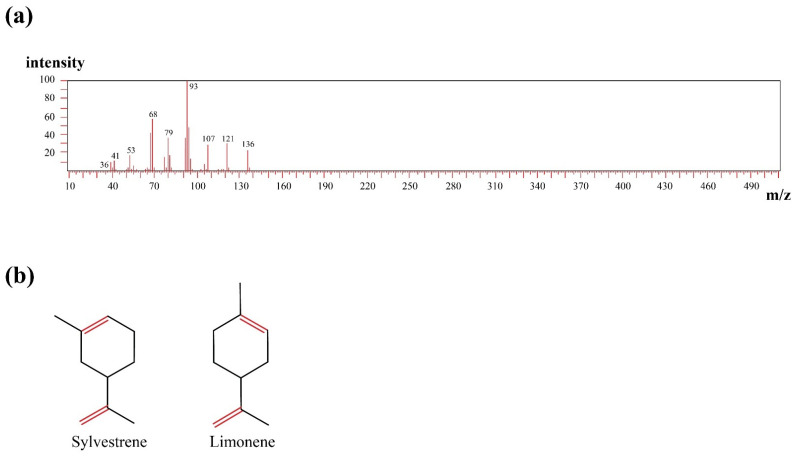
Mass spectrum of sylvestrene in (**a**) and chemical structures of sylvestrene and limonene in (**b**).

Previous reports showed that carvone and limonene are the major constituents but that was misidentified due to the high similarity between mass spectra of both sylvestrene and limonene and close retention index and chemical structure [40]. Sylvestrene’s calculated (exact) mass is 136.23404 *m*/*z*, however, this cannot differentiate it from its positional isomer limonene as we used hard ionization mass in the current study (Figure 2). Testing the antioxidant activity of caraway essential oil using DPPH and ABTS methods revealed strong antioxidant activity with IC50 60 ± 1.17 and 48 ± 0.97 mg/mL, respectively.

### 2.2. Virulence Attenuating Activity of Caraway Oil

**Figure 3:**
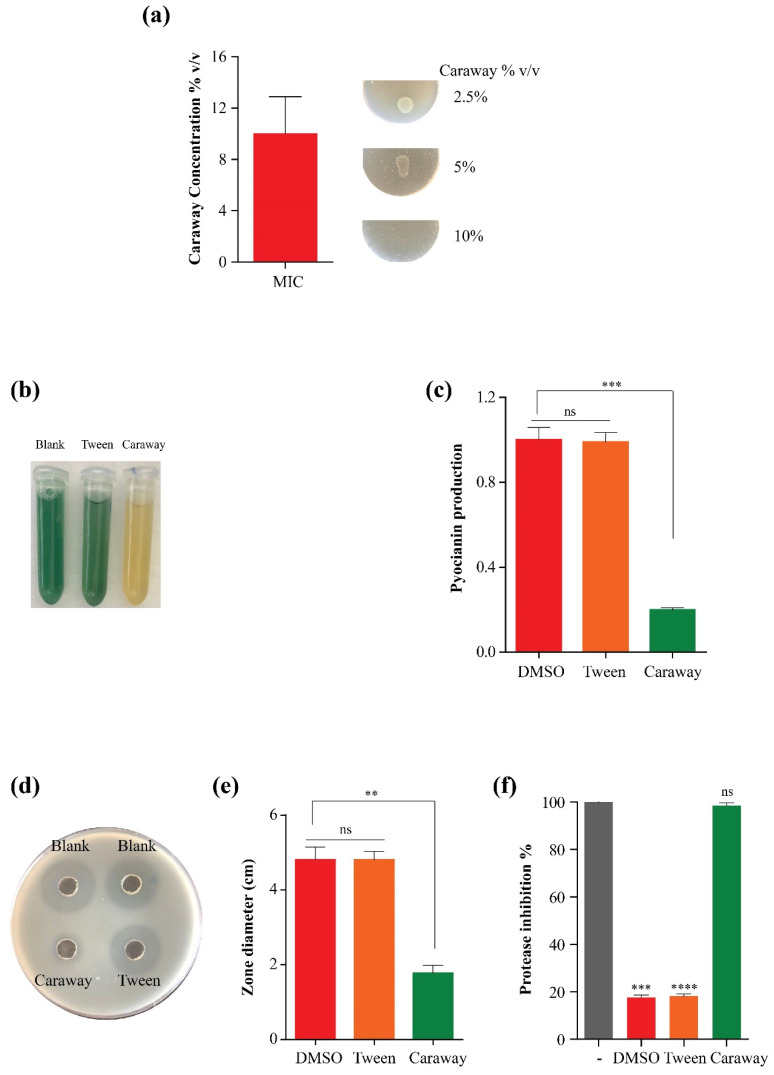
Effect of caraway oil on *P. aeruginosa* growth and virulence. (**a**): Minimum inhibitory concentration (MIC) of the oil, (**b**,**c**): Pyocyanin inhibition by caraway oil, (**d**,**e**): Protease production inhibition by caraway oil using skim milk agar method. (**f**): Protease inhibition assay using modified skimmed milk broth, supernatants from different cultures with or without caraway essential oil were incubated with skimmed milk 1.25% as a negative control for no protease activity, one tube with no supernatants was prepared and denoted as (-). **, ***, and ****: significant change in caraway oil-treated group relative to untreated and Tween-treated groups at *p* < 0.05, ns denotes no significance.

Caraway oil solubilized by Tween (2%) was able to inhibit the growth of *P. aeruginosa* PAO1 strain at a concentration of 10% *v*/*v* (91 mg/mL), we then selected a sub-MIC of 1% *v*/*v* (9.1 mg/mL) which corresponds to 1/10th of the MIC to examine the anti-virulence activity of the oil (Figure 3a).

Pyocyanin (bluish-green pigment) is a characteristic virulence factor and one of the most observable phenotypes of *P. aeruginosa*. Pyocyanin production was dramatically compromised by culturing PAO1 strain in presence of 1% *v*/*v* caraway essential oil. PAO1 strain grown in presence of caraway oil lost 78 to 88% of pyocyanin pigment compared to a control that was cultured in media without the oil. To exclude the possible effect of the solubilizing agent on pigment production, we cultured the PAO1 strain in presence of 2% Tween-20, and there was no significant effect on the pigment production compared to the control (Figure 3b,c).

Next, the ability of caraway oil in sub-MIC to inhibit the virulence enzyme protease was evaluated using the skim milk agar assay. Supernatants were isolated from bacterial cultures that were grown in media supplemented with 1% *v*/*v* caraway oil solubilized with Tween-20 and control cultures to evaluate the bacterial protease activity. Caraway oil exhibited a striking inhibition of the protease activity, a four-fold reduction in the diameter of the clear zone around the well was calculated in the case of caraway oil-treated supernatants compared to controls with or without the solubilizing agent whereas control cultures displayed proficient proteolytic activities of the supernatants (Figure 3d,e). To analyze the inhibition of protease activity in a quantitative manner, we used the modified skimmed milk broth to assay the protease activity of supernatants from caraway-treated cultures or control cultures, caraway oil treatment caused 97 to 100% inhibition of protease activity (Figure 3f).

### 2.3. Caraway Oil Disrupts the Biofilm and Suppresses Quorum Sensing Activity

**Figure 4:**
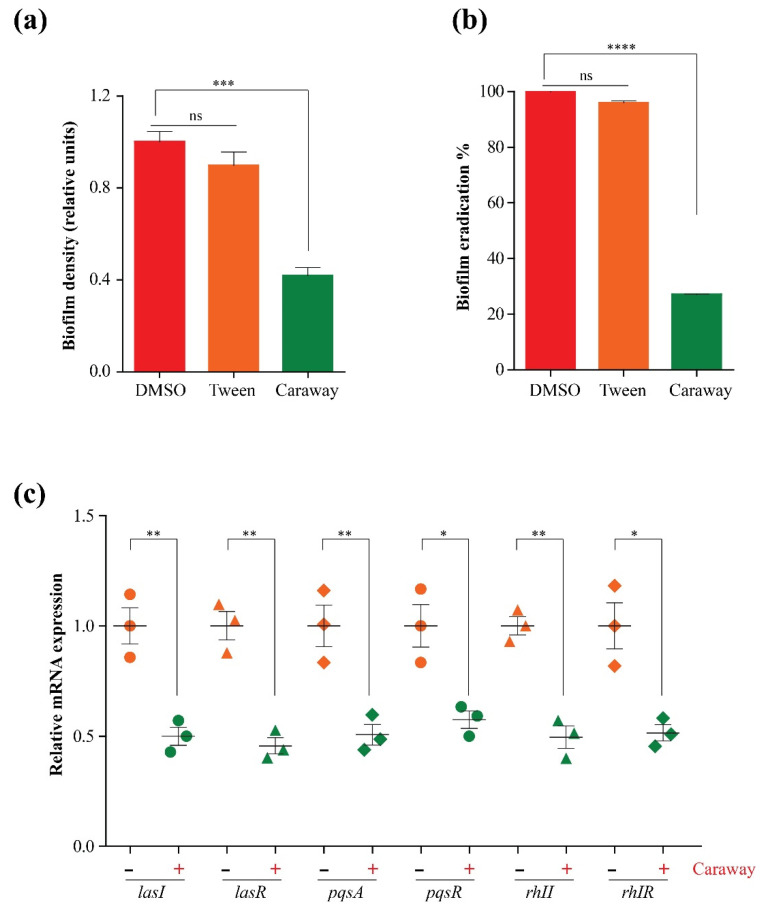
Effect of caraway oil on *P. aeruginosa* biofilm formation and in vitro biofilm eradication (**a**,**b**). qPCR analysis of quorum sensing genes expression in presence or absence of caraway essential oil (**c**), *RopD* was employed as a housekeeping gene. *, **, ***, and ****: significant change in caraway oil-treated group relative to untreated group at *p* < 0.05, ns denotes no significance.

The anti-biofilm activity of caraway oil was tested by incorporation of a sub-MIC dose of caraway oil into PAO1 cultures. The oil led to a remarkable loss of biofilm formation. Caraway oil disrupted the ability of bacterial cells to adhere to the surface and constitute biofilms, in comparison to controls that were cultured in absence of caraway oil, the oil-treated cultures demonstrated 60–72% loss of their biofilm (Figure 4a); moreover, we tested the in vitro biofilm eradication capacity of caraway essential oil, treating the stained bacterial biofilms with caraway essential oil (1% *v*/*v*) removed 72 to 73% of the biofilm denoting powerful biofilm eradication (Figure 4b).

Finally, the effect of caraway oil on quorum-sensing genes was investigated using qPCR. The analysis involved the measurement of expression profiles for six essential genes (*LasI*, *LasR*, *Rhll*, *RhlR*, *PqsA*, and *PqsR*) in the quorum sensing machinery. Our findings demonstrated an abrupt reduction (up to 60%) in the expression of the inspected genes when compared to controls that were cultured in absence of caraway oil (Figure 4c). 

## 3. Discussion

Biofilm formation is a major driver of antibiotic resistance in bacterial communities, and bacterial cells embedded in the biofilm are up to 1000 times more resistant than cells in suspension because biofilm represents a physical barrier against antibiotic penetration [16]. Bacterial biofilm is a product of quorum sensing activation [41], the upstream signaling system supervising cell communication inside the dense microenvironment of biofilm and regulating biofilm formation, in general, is quorum sensing [21]. Recent strategies to challenge antimicrobial resistance focus on the interference with the resistance mechanisms, the virulence factors of the microbes, or quorum sensing this can lead to impairment of cell-to-cell contact, perturbation in bacterial biofilm formation, bacterial motility, and virulence factors such as pyocyanin, protease, and hemolytic ability [42,43,44,45]. 

Researchers are actively working on screening or designing specific compounds that can efficiently inhibit biofilm or suppress bacterial quorum sensing, the rate-limiting step in this approach is the toxicity of most of the developed quorum sensing inhibitors in the clinical stage [46]. To overcome this concern, researchers thought about using natural agents as an alternative way for biofilm disruption or quorum sensing inhibition [8,47]. Essential oils constitute one of these natural agents that could act as antibiofilm as well as quorum sensing inhibitors, for example, essential oils from cassia, balsam Peru, and red thyme are powerful anti-biofilm agents [26,48], marjoram, thyme, sage, rosemary, pignut, lavender, and caraway oils were reported to exert anti-quorum sensing activity [49].

In our study, we chemically defined the components of caraway essential oil using GC-MS, and 13 secondary metabolites were identified representing different hydrocarbons and oxygenated and non-oxygenated terpenes. Although carvone dominated the chromatogram as previously reported, sylvestrene was identified as the second major constituent in the oil, and previous reports showed that it was limonene [29]. Moreover, the current analysis showed a series of long-chain alkanes including nonacosane, triacontane, and unitriacontane, and alkenes such as triacontene, tetratriacontane, and pentatriacontene that were not identified before in caraway oil constituents. These hydrocarbons were reported as antimicrobial agents [50].

Previous studies on caraway oil showed anti-quorum sensing activity against *Vibrio* and *Klebsiella* [31,51]. *P. aeruginosa* is a Gram-negative bacteria armed with arsenals of virulence factors and substantial capability to form biofilm. *Apiaceae* plants have been reported to inhibit virulence factors in *P. aeruginosa* [52]. No studies have been conducted to evaluate the effect of caraway essential oil on the different virulence factors in *P. aeruginosa* including biofilm and quorum sensing. 

Here, we identified that caraway essential oil at a concentration of 10% *v/v* was able to kill *P. aeruginosa* PAO1, a low concentration of the oil (1/10th MIC) with no noticeable effect on *P. aeruginosa* PAO1 viability was used in this sub-inhibitory concentration to evaluate the anti-virulence, anti-biofilm, and anti-quorum sensing activities. The oil was able to disrupt biofilm formation at a sub-MIC (1% *v/v*). Moreover, the signaling molecule and characteristic pigment pyocyanin which is essential for bacterial biofilm formation, bacterial cell fitness, and gene expression [53] was dramatically inhibited to minimal levels of 12–22% of control when cultured in the presence of 1% caraway essential oil. Similarly, the protease enzyme which is essential for anchoring host cells via its hydrolytic activity [54] was completely blocked. The reduction of bacterial protease activity constitutes a good sign for overcoming bacterial spreading [45]. Finally, real-time monitoring of quorum sensing gene clusters, which control bacterial motility and biofilm formation [45,55] using qPCR revealed clear and significant negative effects of caraway essential oil at a sub-MIC dose (1%) on quorum sensing gene circuits on the transcriptional level. Taken together, we represent caraway essential oil as a potential virulence-mitigating agent of natural origin against *P. aeruginosa*.

## 4. Materials and Methods

### 4.1. Plant Material and Preparation of Oil

Two kilograms of *C. carvi* fruits were purchased from a commercial market in Zagazig City, Sharika, Egypt. The fruits were soaked in boiled distilled water for one hour and then hydro-distilled using Clevenger-type apparatus for 4 h to afford 20 mL (extraction yield 1%) that were kept in a dark vial at the freezer until further analysis. The oil was characterized by an aromatic odor and a pale yellow color.

### 4.2. GC/MS Analysis 

The oil was analyzed using a Shimadzu GC/MS-QP2020 (Kyoto, Japan) coupled with Rtx-1MS fused bonded column (30 m length, 0.25 mm internal diameter, and 0.25 μm film thickness, Restek, USA) according to [56,57] with few changes. Briefly, the initial oven temperature was set at 50 °C for 3 min, after that the temperature was increased to 300 °C at a rate of 5 °C/min. Finally, the temperature was held isothermal at 300 °C for 10 min. The injector temperature was adjusted to 280 °C and helium was used as carrier gas with a flow rate of 1.37 mL/min. Mass spectra were acquired utilizing filament emission current (equipment current) of 60 mA, ionization voltage of 70 eV, and ion source temperature at 220 °C. The oil was diluted to 1% *v*/*v* and injected at a split ratio of 1:30. Retention indices (RI) of the isolated components were calculated with respect to a set of standard n-alkanes that were analyzed separately under the same chromatographic conditions.

### 4.3. Antioxidant Activity Estimation

2,2-diphenyl-1-picryl-hydrazyl-hydrate (DPPH)- radical scavenging activity [58], and 2,2′-azino-bis(3-ethylbenzothiazoline-6-sulfonate) (ABTS)- radical scavenging activity [59] tests were used in the estimation of caraway essential oil antioxidant activity:

For the DPPH assay, 250 mL of a DPPH-methanolic solution (0.2 mM) was mixed with 1000 mL of essential oil at different concentrations (10, 20, 30, 40, 50, 60, 70, 80, 90, and 100 mg/mL). The mixture was left in the dark at room temperature for 30 min. The absorbance was measured at 515 nm, and the scavenging activity (RSA%) against DPPH radicals was calculated using the following equation:

RSA (%) = [(Absorbance of control − Absorbance of test)/Absorbance of control] × 100

IC50 values represent the essential oil scavenging 50% of DPPH radicals.

For the ABTS assay, ABTS was generated by mixing a 7 mM of ABTS at pH 7.4 (5 mM NaH_2_PO_4_, 5 mM Na_2_HPO_4_, and 154 mM NaCl) with 2.5 mM Potassium Persulfate (final concentration) followed by storage in the dark at room temperature for 16 h before use. The mixture was diluted with ethanol to give an absorbance of 0.70 ± 0.02 units at 734 nm using a spectrophotometer. An amount of 100 μL of essential oil at different concentrations (10, 20, 30, 40, 50, 60, 70, 80, 90, and 100 mg/mL) was allowed to react with fresh ABTS solution (900 μL), and then the absorbance was measured 6 min after initial mixing. The capacity of free radical scavenging was expressed by IC50 (mg/L) values, which represents the concentration required to scavenge 50% of ABTS radicals. The capacity of free radical scavenging IC50 was determined using the same equation previously used for the DPPH method. 

### 4.4. Bacterial Strain, Media, and Chemicals

The used *P. aeruginosa* PAO1 strain was obtained from the Department of Microbiology, Faculty of Pharmacy, Mansoura University. Microbiological media, Mueller Hinton (MH) broth, Tryptone soya broth (TSB) and agar, and Luria Bertani (LB) broth and agar were purchased from Oxoid (Hampshire, UK). All chemicals were of pharmaceutical grade.

For each experiment, caraway oil was solubilized in the media of interest using 2% Tween-20 in sub-MIC. Control groups from bacterial culture were prepared without caraway oil in presence and absence of the solubilizing agent (Tween-20). 

### 4.5. Minimum Inhibitory Concentration (MIC) Determination

Agar dilution method was used to determine the MIC of caraway oil, 2-fold serial dilutions of caraway oil starting from 20% *v*/*v* in DMSO were made in Mueller–Hinton agar plates using 2% Tween. An amount of 5 μL of 0.5 Mac Farland PAO1 bacterial suspension was spotted on the surface of the plate after 1/10 dilution, then the plates were left to dry under the hood before being incubated overnight at 37 °C. The MIC was calculated as the lowest concentration of caraway oil that inhibited the visible growth of PAO1 strains.

### 4.6. Assessment of Biofilm Inhibition

The biofilm formation was quantified as previously described [60,61]. Briefly, PAO1 overnight cultures in TSB were prepared and diluted with TSB to an optical density OD600 nm of 0.4. Aliquots (10 µL) of the optically adjusted PAO1 suspensions were added each to 10 mL of fresh TSB with caraway oil (1% *v*/*v*) solubilized in Tween or with the solubilizing agent alone or without both of them. The plates were incubated for 24 h at 37 °C. The planktonic cells were aspirated, and the plates were washed 3 times and then left to dry. The adherent bacterial cells were fixed for 25 min with methanol, followed by staining with crystal violet (1%) for another 20 min. The excessive dye was washed, the adhered dye was dissolved in glacial acetic acid (33%), and the absorbance was measured at 590 nm using a Biotek Spectrofluorometer (Biotek, Winooski, VT, USA). The test was made in triplicate and the absorbance of caraway oil-treated PAO1 was shown as mean ± standard error of percentage change from untreated controls.

### 4.7. Biofilm Eradication Assay

Standard strain *P. aeruginosa* ATCC 27853 was grown under ideal conditions to form biofilm as described in [62], biofilms were fixed and stained as previously stated, then tubes containing the formed biofilm were treated with 1% *v*/*v* caraway oil (test) or with solvent only (control), biofilm then washed and dissolved in glacial acetic acid (33%), and the absorbance was measured at 590 nm. To calculate the biofilm eradication percentage (%);

Biofilm eradication % = Absorbance of control at OD590 nm − Absorbance of test at OD590 nm/Absorbance of control at OD590 nm × 100

### 4.8. Assessment of Pyocyanin Production

The ability of caraway oil to reduce pyocyanin production was estimated as described earlier [60,63]. PAO1 overnight cultures were prepared and diluted in LB broth to 600 nm optical density (0.4). One ml LB broth tubes containing caraway oil (1% *v*/*v*) solubilized with Tween or with the solubilizing agent alone or without both of them were inoculated with 10 μL of the bacterial suspensions and incubated at 37 °C for 48 h. The tubes were centrifuged at 9500× *g* for 10 min, and the pyocyanin in the supernatant was spectrophotometrically measured at 691 nm by a Biotek Spectrofluorometer (Biotek, Winooski, VT, USA). The test was conducted in triplicate, and the stain absorbance in the presence of caraway oil or the solubilizing agent alone was expressed as mean ± standard error of percentage change from untreated PAO1 controls.

### 4.9. Evaluation of Protease Activity

In order to evaluate the inhibitory effect of caraway oil on protease activity, the skim milk agar method was employed as described [60,64]. PAO1 overnight cultures in LB broth with caraway oil (1% *v*/*v*) solubilized with Tween or with the solubilizing agent alone or without both of them were centrifuged at 9500× *g* for 20 min. Aliquots (100 µL) of supernatants were added to the wells made in 5% skim milk agar plates, incubated at 37 °C overnight, and the formed clear zones around the wells were measured. The test was performed in triplicate, the obtained results were presented as mean ± standard error of percentage change from untreated controls. 

### 4.10. Modified Skimmed Milk Broth

*P. aeruginosa* ATCC 27853 overnight cultures in MHB with and without 1% *v*/*v* of caraway oil were centrifuged to isolate the supernatants. An amount of 500 μL of supernatants was incubated with 1 mL skimmed milk (1.25%) for 1 h at 37 °C according to [65]. The decrease in optical density of skimmed milk due to protease activity was estimated at 600 nm.

### 4.11. Evaluation of Expression of Quorum Sensing Encoding Genes

The ability of caraway oil to interfere with the expression of quorum sensing genes was assessed by RNA extraction from PAO1 cultures treated and untreated with caraway oil (1% *v*/*v*) solubilized with Tween [60,64]. Briefly, caraway oil-treated and untreated PAO1 cultures were collected by centrifugation (7000 rpm for 10 min at 4 °C). The formed pellets were re-suspended in Tris-EDTA buffer (100 μL) provided with lysozyme and kept at 25 °C for 5 min. Bacterial pellets were lysed by RNA lysis buffer, and total RNA was isolated and purified using GeneJET RNA Purification Kit protocol (Thermoscientific, Waltham, MA, USA). DNase was used to remove residual chromosomal DNA. Finally, RNA concentrations were measured by NanoDrop ND-1000 spectrophotometer and stored at −70 °C until use.

The primers used to evaluate the relative quorum sensing genes’ expression in PAO1 strains by q-PCR were previously listed (Table 2) [55]. The cDNA was obtained using a cDNA Reverse Transcriptase kit (Applied Biosystem, Beverly, MA, USA) that was amplified by the PCR Master Kit Syber Green I (Fermentas) employing the Step One instrument (Applied Biosystem, Beverly, MA, USA). The PCR amplification steps were 10 min/95 °C followed by 40 repeated cycles of 20 s/95 °C, 20 s/62 °C, and 65 s/72 °C. The housekeeping ropD gene was used as the reference gene to normalize the expression of quorum sensing genes, and the relative gene expression was calculated by the comparative threshold cycle (ΔΔCt) method, as described earlier [60,66,67,68]. The experiment was completed in triplicate, and the expression of caraway oil-treated bacterial genes was presented as mean relative to untreated PAO1 controls.

### 4.12. Statistical Analysis

Data are displayed as means ± standard errors of means (SEM) using GraphPad prism 5.0.1 software (GraphPad Software, San Diego, CA, USA). Statistical significance was analyzed by Student’s *t*-test. The probability value (*p* < 0.05) was considered as the level of significance.

## 5. Conclusions

Caraway essential oil was analyzed by GC-MS and thirteen compounds were identified. The oil showed strong antioxidant activity. Microbiological examination demonstrated that caraway essential oil actively impairs *P. aeruginosa* virulence via blockade of pyocyanin production, compromising protease activity, disruption of biofilm formation, and significant capacity to eradicate biofilm in vitro. Measurement of quorum sensing genes’ expression reflected extensive downregulation. Our study spots light on the importance of caraway essential oil as a virulence attenuating agent.

## Figures and Tables

**Table 1:** Essential oil composition of *C. carvi* fruits using GC-MS analysis.

No.	Compounds	Retention Time (min)	Retention Index	Relative Abundance (%)
1	*p*-cymene	9.995	1,007	0.5
2	Sylvestrene	10.335	1,018	14.8
3	cis Dihydrocarvone	14.960	1,164	0.4
4	trans Dihydrocarvone	15.155	1,170	0.6
5	Carveol	16.010	1,198	0.5
6	Carvone	16.530	1,215	63.7
7	Perilla aldehyde	17.195	1,238	0.4
8	Nonacosane	52.385	3,037	0.7
9	Triacontane	52.775	3,066	1.1
10	Unitriacontane	54.580	3,198	0.4
11	Triacontene	56.545	3,342	0.8
12	Tetratriacontene	57.415	3,405	0.8
13	Pentatriacontene	59.015	3,522	0.7

**Table 2:** Sequence of qPCR primers.

Quorum Sensing Gene	Primer Sequence (5′-3′)
**RopD F** **RopD R**	CGAACTGCTTGCCGACTT GCGAGAGCCTCAAGGATAC
**LasI F** **LasI R**	CGCACATCTGGGAACTCA CGGCACGGATCATCATCT
**LasR F** **LasR R**	CTGTGGATGCTCAAGGACTAC AACTGGTCTTGCCGATGG
**RhlI F** **RhlI R**	GTAGCGGGTTTGCGGATG CGGCATCAGGTCTTCATCG
**RhlR F** **RhlR R**	GCCAGCGTCTTGTTCGG CGGTCTGCCTGAGCCATC
**PqsA F** **PqsA R**	GACCGGCTGTATTCGATTC GCTGAACCAGGGAAAGAAC
**PqsR F** **PqsR R**	CTGATCTGCCGGTAATTGG ATCGACGAGGAACTGAAGA

## Data Availability

Not applicable.

## Sample Availability

Samples of the compounds are not available from the authors.
